# Craniospinal irradiation for leptomeningeal metastasis of solid tumors: survival analysis and prognostic factors

**DOI:** 10.1093/jrr/rrae059

**Published:** 2024-08-17

**Authors:** Kazuya Takeda, Rei Umezawa, Takaya Yamamoto, Noriyoshi Takahashi, Keiichi Jingu

**Affiliations:** Department of Radiation Oncology, Tohoku University Graduate School of Medicine, 1-1 Seiryo-machi, Aoba-ku, Sendai, Miyagi 980-8574, Japan; Department of Radiation Oncology, South Miyagi Medical Center, 38-1 Nishi, Ogawara, Shibata, Miyagi 989-1253, Japan; Department of Radiation Oncology, Tohoku University Graduate School of Medicine, 1-1 Seiryo-machi, Aoba-ku, Sendai, Miyagi 980-8574, Japan; Department of Radiation Oncology, Tohoku University Graduate School of Medicine, 1-1 Seiryo-machi, Aoba-ku, Sendai, Miyagi 980-8574, Japan; Department of Radiation Oncology, Tohoku University Graduate School of Medicine, 1-1 Seiryo-machi, Aoba-ku, Sendai, Miyagi 980-8574, Japan; Department of Radiation Oncology, Tohoku University Graduate School of Medicine, 1-1 Seiryo-machi, Aoba-ku, Sendai, Miyagi 980-8574, Japan

**Keywords:** craniospinal irradiation, leptomeningeal metastasis, lymphopenia, systemic inflammatory index (SII)

## Abstract

We conducted a study to examine the treatment outcomes and prognostic factors for patients who underwent craniospinal irradiation (CSI) for leptomeningeal metastasis of solid tumors. This retrospective study included patients who received CSI for leptomeningeal metastasis at a single institute between 2010 and 2021. Data from clinical records and the radiation information system were obtained and analyzed. A total of 25 patients were included in the study. Eighteen patients (72%) completed the scheduled CSI. The median overall survival (OS) period was 4.8 months (95% confidence interval (CI): 3.2–10.0 months). Symptom relief was achieved in four out of 23 symptomatic patients (17%). Non-hematological adverse events occurred in 12 patients (48%), with 1 patient (4%) developing Grade 3 bacterial meningitis and the other patients having Grade 1–2 events. Twenty patients (80%) had hematological adverse events of Grade 3 or higher. Grade 4 hematologic toxicities occurred in 3 patients (12%) due to neutropenia and in 11 patients (44%) due to lymphopenia. In multivariate Cox regression analysis, the systemic immune-inflammation index (SII) was identified as the only significant parameter for predicting OS. The median OS periods for patients with SII < 607 and SII ≥ 607 were 6.1 and 2.1 months, respectively (*P* = 0.003). In conclusion, this study showed the treatment outcomes of CSI for leptomeningeal metastasis of solid tumors. It was shown that a high baseline SII was associated with shorter OS after CSI. The findings will contribute to the evaluation of prognosis after CSI.

## BACKGROUND

Leptomeningeal metastasis is detected clinically in 5–8% of patients with solid tumors and is related to neurological symptoms and poor survival outcomes [1]. Treatment strategy for diffuse leptomeningeal metastasis includes intrathecal chemotherapy, systemic chemotherapy and craniospinal irradiation (CSI). These modalities are applied solely or in combination, showing a modestly improved overall survival (OS) period of ~2 to 4 months, compared with that of 4 to 6 weeks in untreated cases [2]. There have been only a few reports on the use of CSI monotherapy for leptomeningeal metastasis of solid tumors [3, 4]. Those reports showed that 28–53% of patients experienced symptom improvement with median OS periods of 3.4–4.4 months. However, only 53–80% of the patients completed CSI, and ~30% of the patients experienced Grade 3 or higher acute hematologic toxicities. Due to the limited efficacy and high toxicity, the use of CSI is not currently established in treatment guidelines [5].

Investigation of prognostic factors in this patient population may help to identify patients who can benefit from CSI without severe adverse events. Disease distribution and patients’ general condition assessed by ECOG Performance Status (PS) and Karnofsky performance score (KPS) have been shown to predict survival outcomes in this population [3, 4]. Additionally, blood test parameters such as serum albumin level, hemoglobin concentration and ratios of different blood subfractions including neutrophil-to-lymphocyte ratio (NLR), platelet-to-lymphocyte ratio (PLR) and systemic immune-inflammation index (SII) have been reported to be useful for predicting OS in cancer patients [6–8]. However, there has been no study in which the predictive values of these parameters in patients with leptomeningeal metastasis were investigated.

In this study, we conducted a retrospective analysis of patients with leptomeningeal metastasis who received CSI. The aim of this study was to determine survival outcomes and prognostic factors as well as the treatment’s efficacy and acute adverse events for future treatment decision-making.

## MATERIALS AND METHODS

### Study participants

This study received approval from the Institutional Review Board of Tohoku University Graduate School of Medicine (2021-1-1073). This retrospective study included patients who underwent CSI for leptomeningeal metastasis between January 2010 and December 2021. Leptomeningeal metastasis was diagnosed using magnetic resonance imaging (MRI) with gadolinium enhancement or cerebrospinal fluid (CSF) testing. The decision to perform CSI was made by the attending physician on a case-by-case basis. In the majority of cases, CSI was considered when imaging or CSF studies revealed disseminated lesions in the spinal cord region and when neurological symptoms were present. Each patient was classified based on EANO-ESMO guidelines as follows [5]. Type I: positive CSF cytology or biopsy; Type II: clinical findings and neuroimaging only. Type A: leptomeningeal metastasis with typical linear MRI abnormalities; Type B: leptomeningeal metastasis with nodular disease only; Type C: leptomeningeal metastasis with both linear and nodular disease and Type D: leptomeningeal metastasis without MRI abnormalities except possibly hydrocephalus. Patients with primary central nervous system (CNS) tumors or hematological malignancies were excluded. CSI was defined as irradiation of the entire brain and spinal canal. If patients had a history of whole brain (WB) irradiation, whole spinal-only irradiation was also included as CSI in this study. We divided these cases into sequential CSI and separate CSI as follows: sequential CSI was defined as whole spinal irradiation (SI) administered sequentially after WB irradiation, and separate CSI was defined as whole SI administered after WB irradiation with an interval period. For such cases, the start of SI was considered the beginning of CSI. Modifications in radiation fields and dose prescriptions considering past irradiation history were allowed if the total treatment course could be clinically recognized as CSI. Clinical information was obtained from hospital records and the radiation information system. Symptom relief was documented if there was a subjective or objective improvement in symptoms present at the start of CSI. Non-hematologic adverse events referred to newly developed symptoms after the initiation of CSI. The severity of toxicities was graded according to the Common Terminology Criteria for Adverse Events (CTCAE) version 5.0.

### Radiotherapy procedure

The patients were positioned either in the supine or prone position and underwent 3D treatment planning with CT simulators. CSI was delivered using 4, 6 or 10 MV X-ray beams generated by a linear accelerator (Clinac iX or TrueBeam, Varian Medical Systems, Inc., Palo Alto, CA). The clinical target volume was defined as the whole brain and spinal canal, with an appropriate margin added. In beam arrangement, WB irradiation with horizontal beams was combined with whole spinal canal irradiation with one or two dorsal beams. The dose in the junction area was averaged using the moving junction technique. The prescribed treatment dose was 30 Gy delivered in 15 fractions, with a dose of 2 Gy per fraction. Adjustments were made as necessary based on the individual characteristics of each patient. Boost irradiation was administered to localized disease sites when deemed necessary. Patients who had previously undergone WB irradiation only received irradiation of the entire spinal canal. In some cases, sequential whole SI was performed after WB irradiation due to the diagnosis of leptomeningeal metastasis in the spinal canal, as revealed by MRI during the period of whole brain radiotherapy.

### Blood test data

In the blood test data, we investigated neutrophil count, lymphocyte count, platelet count, hemoglobin concentration and serum albumin concentration. We calculated the NLR, PLR and SII as follows: NLR = neutrophil count / lymphocyte count, PLR = platelet count / lymphocyte count and SII = (neutrophil count × platelet count) / lymphocyte count. Baseline data were defined as the most recent data available at the start of CSI. For hematological parameters, the nadir was defined as the lowest value within a 12-week period starting from the day after the initiation of CSI. Hematologic toxicities were graded according to CTCAE version 5.0. The following criteria were used to assess Grade 1 events: neutropenia: < 2.0 × 10^3^/μl, lymphopenia: <1.0 × 10^3^/μl, thrombocytopenia: <150 × 10^3^/μl and anemia: < 12 g/dl. When assessing anemia, only hemoglobin concentration was considered. For the calculation, the values were assumed to remain constant until the next value was obtained.

### Statistical analysis

For the baseline analysis, each variable was presented as a number with a percentage or a median with an interquartile range or an entire range, as appropriate. OS was calculated as the period from the start of CSI to the patient’s death or the last follow-up. Survival curves were plotted and evaluated using the Kaplan–Meier method.

For visualization, CTCAE grades of neutropenia, lymphopenia, thrombocytopenia and anemia at baseline and the nadir were plotted. For patients who survived longer than 12 weeks, changes in hematological parameters over time were evaluated. In this analysis, the baseline values and the lowest values in each 2-week interval after the start of CSI up to 12 weeks were considered. To visualize the time course of changes, the mean, range and interquartile range were plotted for each period. Spline curves with λ = 0.05 were used to approximate the curves.

In evaluating predictive factors for OS, continuous variables were cut off at the median and treated as binary variables. The Cox proportional-hazards model was used to identify predictive factors for patients’ OS. Statistical significance was determined by a *P*-value of <0.05. In the multivariate analysis, factors with a *P*-value of < 0.05 in the univariate analysis were included with consideration of the small number of the events. The log-rank test was used to evaluate the difference between survival curves.

All statistical analyses were performed using JMP Pro 17.1.0 (JMP Statistical Discovery LLC, NC) and R 4.2.2 (The R Foundation for Statistical Computing Platform, Vienna, Austria).

## RESULTS

### Patient characteristics

A total of 25 patients were included in this study. Table 1 shows the characteristics of the patients. Breast cancer was the most common primary disease (16 (64%) of the patients). For the diagnosis of leptomeningeal metastasis, all of the 25 patients received MRI, while only two patients (8%) received a pathological diagnosis through CSF testing. MRI revealed that 16 patients (56%) had a linear pattern, while seven (28%) had a nodular pattern. Two patients (8%) had a history of using granulocyte colony-stimulating factor (G-CSF). Seventeen patients (68%) underwent simultaneous whole brain and whole spine irradiation, whereas eight patients (32%) received whole spine irradiation after whole brain irradiation. Of the eight patients, four patients (16%) received whole spine irradiation immediately after whole brain irradiation and four patients (16%) had an interval between spine and brain irradiation with a median of 89.5 days (range: 43–151 days). The median dose and fractionation were 30 Gy in 15 fractions, and it was the most commonly used dose fractionation (eight cases, 32%). Boost irradiation after CSI for dominant spinal lesions was added in 3 patients (12%), with 7.5–16 Gy in 3–8 fractions being administered. Thirteen patients (52%) had a history of radiotherapy (RT) and six patients (24%) had direct irradiation for the brain or spinal cord. In those six patients, three patients had a history of SI due to RT for the vertebrae or whole neck, two patients had a history of stereotactic radiosurgery for brain metastases and one patient had both spinal and cranial irradiation.

**Table 1:** Patient characteristics

Characteristic	*N* = 25^a^
Sex	
Female	21 (84%)
Male	4 (16%)
Age [years old]	60 (53, 66)
Primary site	
Breast cancer	16 (64%)
Luminal type	10 (63%)
HER2 enriched	2 (13%)
Triple-negative	3 (19%)
Unknown	1 (6%)
Lung cancer	3 (12%)
Adenocarcinoma	1 (33%)
Small cell carcinoma	2 (67%)
Ovarian cancer	2 (8%)
Others^b^	4 (16%)
Treatment history	
Surgery	20 (80%)
Chemotherapy	24 (100%)
Radiotherapy	13 (52%)
CNS irradiation	6 (24%)
G-CSF use	2 (8%)
Time from diagnosis to CSI [years]	2.7 (1.4, 7.5)
PS	
0	2 (8.0%)
1	7 (28%)
2	11 (44%)
3	2 (8.0%)
4	3 (12%)
Symptom presence	23 (92%)
Neurologic symptom	22 (88%)
Pain	12 (48%)
Diagnosis	
MRI	25 (100%)
PET	1 (4%)
CSF test	2 (8%)
EANO-ESMO Diagnostic criteria	
IA	1 (4%)
ID	1 (4%)
IIA	13 (52%)
IIB	7 (28%)
IIC	3 (12%)
Disease outside CNS	14 (56%)
Irradiation type	
CSI	21 (84%)
SI	4 (16%)
Time to SI from WB	
Simultaneous	17 (68%)
Sequential	4 (16%)
Separate	4 (16%)
Irradiation dose [Gy]	30 (24, 31)
Dose per fraction [Gy]	
1.8	3 (12%)
2.0	17 (68%)
2.5	5 (20%)
Boost irradiation	3 (12%)
Neutrophil count [10^3^/μl]	4.02 (2.72, 4.79)
Lymphocyte count [10^3^/μl]	1.15 (0.89, 1.66)
Hemoglobin [g/dl]	12.5 (11.7, 13.6)
Platelet [10^3^/μl]	231 (176, 283)
Albumin [g/dl]	3.9 (3.6, 4.2)
NLR	2.6 (2.1, 3.6)
PLR	195 (134, 275)
SII	607 (412, 977)

^a^Shown as number with percentage or as median with interquartile range.

^b^Including one patient each with nasopharyngeal cancer, gastric cancer, ureteral cancer and rhabdomyosarcoma. PET = positron emission tomography, CSI = cerebrospinal irradiation, WB = whole brain irradiation.

### Clinical course

**Fig. 1:**
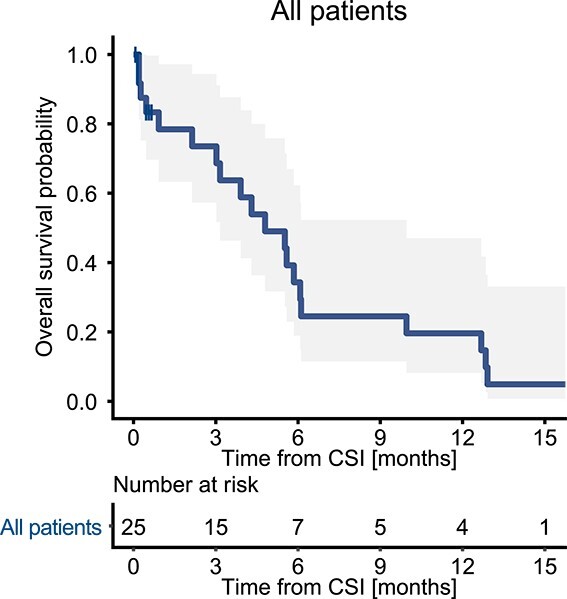
Kaplan–Meier curves of all patients (*n* = 25).

Clinical course, treatment effect and acute toxicities are shown in Table 2. Eighteen (72%) of the patients completed the CSI. Seven patients (28%) could not complete the RT due to disease progression in six patients (24%) and bacterial meningitis in one patient (4%). At the time of the survey, 21 patients (84%) were dead and four patients (16%) were lost to follow-up. The median OS period was 4.8 months (95% confidence interval (CI): 3.2–10.0 months, Fig. 1). The median OS period of the 18 patients who completed the CSI was 5.6 months, and the median OS period for the 7 patients who discontinued CSI was 0.4 months (*P* = 0.02, log-rank test). Of 23 patients who had some symptoms at baseline, four patients (17%) had symptom relief. Two of those patients showed functional improvement, one patient being able to stand with support and one patient having vision improvement. The remaining two patients showed mild improvement in their neurological symptoms including numbness in the leg and facial nerve palsy. The median period from the start of CSI to symptom relief was 18 days (range: 11–26 days). Six patients (24%) continued systemic therapy after CSI, and 19 patients (76%) received the best supportive care.

**Table 2:** Treatment effect and acute toxicities

Characteristic	*N* = 25^a^
Treatment completion	18 (72%)
Treatment after CSI	
Yes	6 (24%)
No	19 (76%)
Presence of baseline symptoms	23 (92%)
Symptom improvement	4 (17%*)
Time to improvement [days]	18 (11–26)
Follow up examination^b^	12 (48%)
Objective improvement	9 (75%*)
Time to improvement [days]	69 (20–97)
Acute non-hematologic toxicity	
Any grade	12 (48%)
Grade 1–2	11 (44%)
Nausea	8 (32%)
Vomit	4 (16%)
Mucositis	6 (24%)
Pain flare	1 (4%)
Others	3 (12%)
Grade 3	1 (4%)
Bacterial meningitis	1 (4%)

^a^Shown as a number with percentage or as a median with range.

^b^For follow-up, 11 patients underwent MRI, while one patient underwent a CSF test. Percentages with an asterisk (*) were calculated based on the number of evaluable patients. CSI = cerebrospinal irradiation.

### Non-hematologic toxicities

After the start of CSI, 12 patients (48%) experienced newly developed symptoms, which were classified as acute adverse events. A Grade 3 event was observed in one patient (4%) who developed bacterial meningitis after 30 Gy of CSI, resulting in treatment discontinuation. The other 11 patients (44%) experienced Grade 1–2 adverse events including malaise, nausea, vomiting, mucositis, hair loss and pain flare. No Grade 4–5 events were observed.

### Hematologic toxicities

**Fig. 2:**
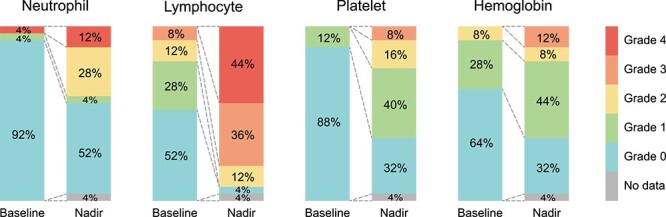
Evaluation of hematological parameters at baseline and nadir in the 12-week period from the start of CSI. Each patient was assessed using CTCAE version 5.0. For assessment of Grade 1 events, the following criteria were used: neutropenia: <2.0 × 10^3^/μl, lymphopenia: <1.0 × 10^3^/μl, thrombocytopenia: <150 × 10^3^/μl, and anemia: < 12 g/dl.

**Fig. 3:**
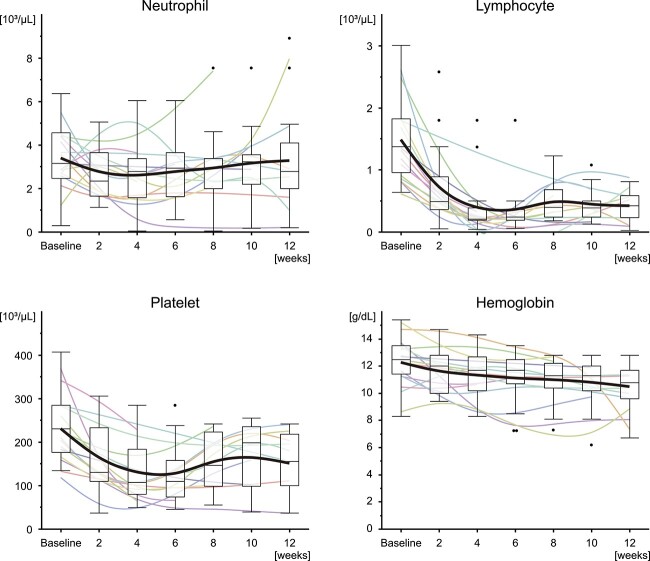
Time courses of changes in hematological parameters. Box plots show the distribution of nadir values for the patients in the last 2 weeks. The bold black curves represent spline curves of the mean values, and pale color curves represent the spline curves of patients.

In the baseline evaluation, data for hematologic parameters within 4 weeks before the start of CSI were available for 23 patients (92%) and data for albumin within 4 weeks before the start of CSI were available for 21 patients (84%). For the remaining patients, the most recent data prior to starting CSI were considered as baseline data. Figure 2 shows the hematologic toxicities at baseline and the nadir in a 12-week period after the start of CSI. Hematologic toxicities of Grade 3 or higher were observed in 20 patients (80%). All of the 20 patients showed Grade 3 or higher lymphocytopenia. Apart from lymphopenia, six patients (24%) showed Grade 3 or higher hematologic toxicities in duplicate, including leukopenia in three (12%), thrombocytopenia in two (8%) and anemia in three patients (12%). Grade 4 hematologic toxicities were observed in 13 patients (52%), including neutropenia in 3 patients (12%) and lymphopenia in 11 patients (44%). One patient (4%) received G-CSF after CSI for the neutropenia. Fifteen patients (60%) were alive and were followed up for 12 weeks after the start of CSI. Figure 3 shows the changes in blood counts over the course of 12 weeks for those 15 patients. Lymphocyte and platelet counts showed an acute decline and gradual recovery following the nadir at ~4 weeks. Hemoglobin concentration showed a trend for a gradual decline over the 12-week period. Neutrophil values varied significantly among patients, with no consistent trend observed.

### Survival analysis

**Fig. 4:**
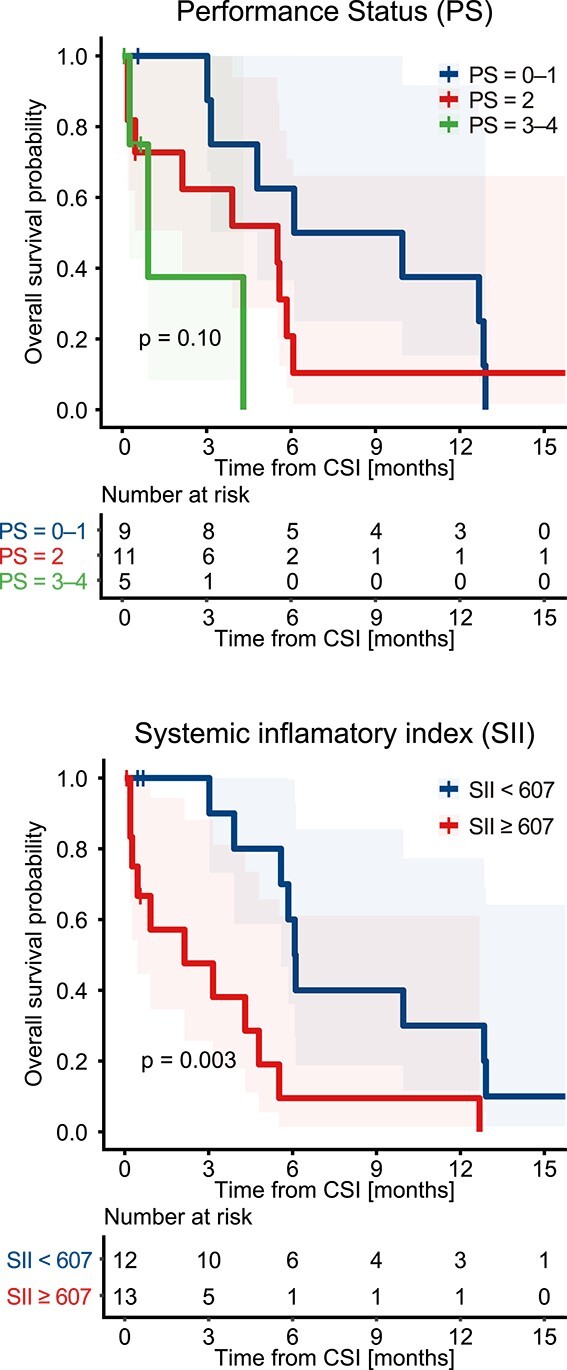
Kaplan–Meier curves divided by PS and the median value of SII. The differences between the groups were tested using the log-rank test.

Table 3 shows the results of Cox regression analysis for investigating predictive factors for OS. Univariate analysis revealed that PS, NLR, PLR and SII at treatment baseline were significant predictors. Since NLR, PLR and SII share the same hematological parameters, the multivariate analysis included SII, which showed the highest hazard ratio (HR)and the smallest *P*-value, along with PS. The multivariate analysis showed that SII was a significant predictor of OS (HR: 4.56; *P* = 0.006). Figure 4 shows the survival curves stratified by PS and the median SII value of 607. The median OS periods for patients with SII < 607 and SII ≥ 607 were 6.1 and 2.1 months, respectively (*P* = 0.003, log-rank test).

**Table 3:** Cox proportional-hazards model regression analysis

		Univariate				Multivariable	
Characteristic	Category	HR	95% CI	*P*-value	HR	95% CI	*P*-value
Sex	Male vs female	0.79	0.23, 2.75	0.7			
Age	≥ 60 years old	0.62	0.26, 1.50	0.3			
Primary disease	Breast vs others	1.01	0.39, 2.60	>0.9			
Treatment history	≥ 3 years	0.88	0.36, 2.13	0.8			
Surgery	Yes vs no	0.71	0.23, 2.19	0.6			
Chemotherapy	Yes vs no	—^a^	—^a^	—^a^			
Radiotherapy	Yes vs no	0.37	0.13, 1.01	0.051			
CNS irradiation	Yes vs no	0.94	0.33, 2.64	0.9			
Performance status	0–1	—	—	—	—	—	—
	2	1.70	0.63, 4.53	0.3	2.31	0.81, 6.58	0.12
	3–4	4.67	1.03, 21.1	**0.046**	2.98	0.66, 13.5	0.2
Any symptoms	Yes vs no	2.49	0.56, 11.1	0.2			
Neurologic symptom	Yes vs no	1.76	0.50, 6.17	0.4			
Pain	Yes vs no	1.25	0.50, 3.15	0.6			
Disease outside CNS	Yes vs no	1.45	0.60, 3.54	0.4			
Diagnostic criteria							
EANO-ESMO diagnostic criteria							
Diagnosis	Type I (pathological)	—	—				
	Type II (clinical)	0.66	0.15, 2.96	0.6			
MRI findings	Type A (linear)	—	—				
	Type B (nodular)	0.51	0.17, 1.48	0.2			
	Type C (both)	0.92	0.25, 3.41	>0.9			
	Type D (negative)	0.57	0.07, 4.58	0.6			
Irradiation type	SI vs CSI	4.28	0.94, 19.6	0.06			
Irradiation dose	≥ 30 Gy	0.85	0.30, 2.39	0.8			
Neutrophil count	≥ 4.02 × 10^3^/μl	1.33	0.54, 3.30	0.5			
Lymphocyte count	≥ 1.15 × 10^3^/μl	0.40	0.15, 1.03	0.06			
Hemoglobin	≥ 12.5 g/dl	0.58	0.24, 1.43	0.2			
Platelet	≥ 231 × 10^3^/μl	1.12	0.46, 2.72	0.8			
Albumin	≥ 3.9 g/dl	1.01	0.41, 2.51	>0.9			
NLR	≥ 2.6	2.98	1.13, 7.83	**0.027**	—^b^	—^b^	—^b^
PLR	≥ 195	3.78	1.40, 10.2	**0.008**	—^b^	—^b^	—^b^
SII	≥ 607	4.10	1.52, 11.1	**0.005**	4.59	1.54, 13.6	**0.006**

Factors with a *P*-value of < 0.05 in the univariate analysis were included in the multivariate analysis. Factors with a *P*-value of < 0.05 are shown in bold. CSI = cerebrospinal irradiation.

^a^Not available because all patients had a history of chemotherapy.

^b^NLR and PLR were excluded due to overlapping with SII in the components.

### Additional analysis for SII

We conducted additional exploratory analyses for baseline SII. Patient characteristics divided by baseline SII value are shown in Supplementary Table 1. Patients with lower baseline SII tended to be older (median age: 64 vs 54 years old, *P* = 0.2) and had better PS (PS 0 or 1: 42% vs 31%, *P* = 0.5), but there was no statistical significance. The treatment completion rates were 83% and 61% in patients with SII < 607 and SII ≥ 607 at baseline, respectively (*P* = 0.38, chi-squared test). As for symptom improvement, 3 of 10 patients with baseline symptoms who had SII < 607 (30%) experienced improvement in their symptoms and 1 of 13 patients who had SII ≥ 607 (8%) had symptom relief (*P* = 0.16, chi-squared test).

## DISCUSSION

In this study, we conducted a retrospective analysis of 25 patients who underwent CSI for leptomeningeal metastasis. The treatment completion rate was 72% with a symptom improvement rate of 17% and a median OS period of 4.8 months. We also conducted a detailed investigation of hematological toxicity following CSI and showed the predictive value of baseline SII for patients’ OS.

CSI has been a treatment option for leptomeningeal metastasis for a long time. Historically, CSI was often administered in combination with intrathecal chemotherapy [9–11]. However, this combined approach was associated with severe myelosuppression and leukoencephalopathy, limiting its overall effectiveness. As a result, radiation monotherapy has received increasing attention in recent years. In two studies that focused on CSI as a radiation monotherapy, median OS periods ranged from 3.4 to 4.4 months and treatment completion rates ranged from 53% to 80% [3, 4]. These findings are consistent with the findings of our study, indicating that CSI is still a highly toxic treatment modality for patients with metastatic diseases. On the other hand, some patients seem to have benefits from CSI including symptom relief and a survival benefit. In this study, 17% of the symptomatic patients had symptom relief, which was relatively lower compared to the rates reported in previous studies, ranging from 28% to 53% [3, 4]. These facts emphasize the importance of understanding adverse events to utilize CSI safely. Considering the low treatment completion rate in limited PS patients (80% in PS 0–2 patients vs 40% in PS 3–4 patients, *P* = 0.11), patients with a reserved general condition may be suitable for CSI.

Despite a high rate of treatment discontinuation, non-hematological adverse events caused by CSI are generally mild. Therefore, it is important to manage hematological toxicities associated with CSI. In this study, we investigated the incidence rates of hematological toxicities and the details of their temporal changes. In neutrophils, red blood cells and thrombocytes, Grade 3 acute cytopenia has been reported to occur in about 30% of patients who receive CSI without concurrent chemotherapy [3, 4], being consistent with our study (six patients, 24%). Neutropenia is a well-recognized adverse event following RT. In our study, 12% of the patients experienced Grade 4 neutropenia and one patient (4%) received G-CSF treatment. Neutropenia can lead to severe infections and requires frequent monitoring. Although thrombocytopenia and anemia are less frequent adverse events, Grade 3 thrombocytopenia and anemia were observed in 8% and 12% of our patients, respectively. Notably, anemia appears to persist and worsen even 12 weeks after CSI, emphasizing the need for regular evaluation. Lymphopenia has received little attention because of the lack of noticeable clinical symptoms in most patients and has not been evaluated in previous studies. In our study, 70% of the patients experienced Grade 3 or higher lymphopenia.

Recently, increasing evidence supports an association between lymphopenia after RT and poor outcomes [12–14]. Although the mechanism by which lymphopenia occurs is not fully understood, irradiation to the bloodstream is believed to primarily affect lymphocytes due to their high radiosensitivity [15]. The severity of lymphopenia after RT is primarily associated with baseline lymphocyte count [16]. In this study, patients in high SII group showed a tendency of lower baseline lymphocyte count and higher grade of lymphopenia after CSI. Baseline SII, which includes lymphocyte count in the calculation formula, seems to be useful in considering lymphopenia after CSI. Another approach for preventing lymphopenia after CSI includes the use of advanced radiation techniques such as intensity-modulated RT (IMRT), volumetric-modulated arc therapy (VMAT) and proton beam therapy shows promise for reducing hematological toxicities by reducing the irradiated volume outside the treatment target. IMRT, including VMAT, has been suggested to be useful in reducing severe acute hematological toxicity in patients with gynecologic and rectal cancer by reducing irradiation outside the treatment target [17, 18]. In recent years, proton therapy has also demonstrated the potential to mitigate lymphocytopenia after broad-field irradiation [19–21]. Yang *et al*. conducted a randomized phase II trial in patients with solid tumor leptomeningeal metastasis in which proton CSI was compared with photon involved-field radiotherapy (IFRT) [22]. That trial met its primary endpoint of prolonged CNS progression-free survival and showed median OS periods of 9.9 and 7.5 months in the proton CSI and photon IFRT groups, respectively. Grade 4 adverse events occurred in only 10% of the patients in the proton CSI group, all of which were lymphopenia. These approaches using advanced irradiation techniques are promising, and further studies are necessary for evaluation.

Lymphopenia has an important role also in prediction of patients’ prognosis at treatment baseline. There have been reports on the potential usefulness of combined parameters involving lymphocyte counts, such as NLR, PLR, and SII, in reflecting systemic immune and inflammatory status and serving as prognostic factors in various cancer treatment scenarios [6, 8, 23]. In this study, we found that baseline SII was an independent prognostic factor for patient survival at baseline. To the best of our knowledge, only one report has previously shown the impact of SII on outcomes in patients with leptomeningeal metastasis from lung cancer [24]. Our study is the first study showing that the impact of SII remains significant in patients who have received CSI. In our study, patients with SII < 607 had longer OS with statistical significance and tendencies for higher rate of treatment completion and higher response rate. Given the relatively long treatment duration (2 to 3 weeks) and high treatment burden associated with CSI, considering pre-treatment parameters, such as the SII, may be helpful in prognostic prediction in determining an optimal treatment strategy for each patient.

Among other baseline parameters, a higher ECOG PS tended to be associated with shorter OS, although statistical significance was not reached (*P* = 0.12). In terms of patient characteristics, 44% of the patients were evaluated to have PS 2. Previous studies have indicated that KPS is a predictor of OS with statistical significance, suggesting that a more detailed evaluation using KPS may be more suitable for this patient population. Regarding the EANO-ESMO diagnostic criteria, patients with Types IIB and IIC, who had non-pathological diagnoses and nodular type lesions, showed worse prognoses compared to patients with Types IIA and IID [25]. Conversely, in our study, patients with type B (nodular pattern) tended to have a lower HR compared to Type A (linear pattern) in survival analysis (HR: 0.51, 95% CI: 0.17–1.48, *P*-value: 0.2). Among our patients, many of those with Type A had diffuse linear lesions around the spinal cord, which could be associated with a poorer prognosis. Pathological and molecular subtypes have also been reported to be associated with patients’ prognosis. Leptomeningeal metastasis patients with breast cancer as the primary disease have been reported to have a longer prognosis than those with lung cancer or melanoma [25]. In the breast cancer population, the triple-negative subtype has been reported to be associated with a poor prognosis [26, 27]. However, in the current study, we were unable to identify a clear relationship between pathological findings and patients’ survival due to the limited sample size.

We recognize some limitations in our study. First, the number of patients was small and patients’ backgrounds were diverse, which made generalizing the results of this study difficult. Second, all patients received conventional 3D conformal RT. As discussed earlier, it would be necessary to compare the efficacy and safety of IMRT or proton beam therapy to those of conventional RT. Third, we were unable to obtain detailed clinical information, such as KPS and time-course changes in non-hematological toxicities, due to limited data availability. Fourth, it remains unclear whether SII can be used as a predictor for symptom relief and prognostic benefits of CSI in each patient because of the retrospective and single-arm study design.

In conclusion, this study provides insights into the treatment outcomes of CSI in patients with leptomeningeal metastasis of solid tumors. We have shown comprehensive hematological data, including the time course of changes in blood cell count, as well as the negative impact of high baseline SII on patients’ OS. These findings will contribute to the evaluation of prognosis and potential hematological toxicities following CSI treatment for individual patients.

## Supplementary Material

TableSup1_20240515_without_highlight_rrae059
