# Supplementary material for: Craniospinal irradiation for leptomeningeal metastasis of solid tumors: survival analysis and prognostic factors
Source: J Radiat Res. 2024 Aug 17;65(5):667–75. doi: 10.1093/jrr/rrae059 (PMC11420835; doi:10.1093/jrr/rrae059)
Supplement: TableSup1_20240515_without_highlight_rrae059 [file tablesup1_20240515_without_highlight_rrae059.docx]

Supplementary table 1. Patient characteristics divided by SII.

| **Characteristic** | | **SII<607**, N = 12^1^ | | **SII≥607**, N = 13^1^ | | **p-value** | |
| --- | --- | --- | --- | --- | --- | --- | --- |
| Sex | |  | |  | | >0.9 | |
| Female | | 10 (83%) | | 11 (85%) | |  | |
| Male | | 2 (17%) | | 2 (15%) | |  | |
| Age [years old] | | 64 (56, 67) | | 54 (51, 64) | | 0.2 | |
| Primary site | |  | |  | | 0.8 | |
| Breast cancer | | 7 (58%) | | 9 (69%) | |  | |
| Luminal type | | 6 (86%) | | 4 (44%) | |  | |
| HER2 enriched | | 1 (14%) | | 1 (11%) | |  | |
| Triple-negative | | 0 (0%) | | 3 (33%) | |  | |
| Unknown | | 0 (0%) | | 1 (11%) | |  | |
| Lung cancer | | 1 (8%) | | 2 (15%) | |  | |
| Adenocarcinoma | | 0 (0%) | | 1 (50%) | |  | |
| Small cell carcinoma | | 1 (100%) | | 1 (50%) | |  | |
| Ovarian cancer | | 1 (8%) | | 1 (8%) | |  | |
| Others | | 3 (25%) | | 1 (8%) | |  | |
| Treatment history | |  | |  | |  | |
| Surgery | | 11 (92%) | | 9 (69%) | | 0.3 | |
| Chemotherapy | | 12 (100%) | | 13 (100%) | |  | |
| Radiotherapy | | 8 (67%) | | 5 (38%) | | 0.2 | |
| CNS irradiation | | 3 (25%) | | 3 (23%) | | >0.9 | |
| G-CSF use | | 1 (8%) | | 1 (8%) | | >0.9 | |
| Time from diagnosis to CSI [years] | | 4.4 (2.5, 9.4) | | 2.1 (1.2, 4.3) | | 0.2 | |
| Performance Status | |  | |  | | 0.5 | |
| 0 | | 2 (17%) | | 0 (0%) | |  | |
| 1 | | 3 (25%) | | 4 (31%) | |  | |
| 2 | | 6 (50%) | | 5 (38%) | |  | |
| 3 | | 0 (0%) | | 2 (15%) | |  | |
| 4 | | 1 (8%) | | 2 (15%) | |  | |
| Symptom presence | | 10 (83%) | | 13 (100%) | | 0.2 | |
| Neurologic symptom | | 9 (75%) | | 13 (100%) | | 0.1 | |
| Pain | | 7 (58%) | | 5 (38%) | | 0.3 | |
| Diagnosis | |  | |  | |  | |
| MRI | | 12 (100%) | | 13 (100%) | | – | |
| PET | | 0 (0%) | | 1 (8%) | | >0.9 | |
| CSF test | | 1 (8%) | | 1 (8%) | | >0.9 | |
| EANO-ESMO Diagnostic criteria | |  | |  | | >0.9 | |
| IA | | 0 (0%) | | 1 (8%) | |  | |
| ID | | 1 (8 %) | | 0 (0%) | |  | |
| IIA | | 6 (50%) | | 7 (54%) | |  | |
| IIB | | 4 (33%) | | 3 (23%) | |  | |
| IIC | | 1 (8%) | | 2 (15%) | |  | |
| Disease outside CNS | | 6 (50%) | | 8 (62%) | | 0.6 | |
| Irradiation type | |  | |  | | >0.9 | |
| CSI | | 10 (83%) | | 11 (85%) | |  | |
| SI | | 2 (17%) | | 2 (15%) | |  | |
| Time to SI from WB | |  | |  | | 0.8 | |
| Simultaneous | | 9 (75%) | | 8 (62%) | |  | |
| Sequential | | 1 (8%) | | 3 (23%) | |  | |
| Separate | | 2 (17%) | | 2 (15%) | |  | |
| Irradiation dose [Gy] | | 30 (29, 36) | | 30 (12, 30) | | 0.06 | |
| Dose per fraction [Gy] | |  | |  | | 0.5 | |
| 1.8 | | 2 (17%) | | 1 (8%) | |  | |
| 2.0 | | 9 (75%) | | 8 (62%) | |  | |
| 2.5 | | 1 (8%) | | 4 (31%) | |  | |
| Boost irradiation | | 1 (8%) | | 2 (15%) | | >0.9 | |
| Neutrophil count [10^3^/μl] | | 3.00 (2.49, 4.09) | | 4.62 (3.23, 5.02) | | **0.03** | |
| Lymphocyte count [10^3^/μl] | | 1.40 (0.97, 2.15) | | 0.95 (0.82, 1.38) | | 0.08 | |
| Hemoglobin [g/dl] | | 12.70 (12.10, 13.30) | | 12.10 (11.40, 13.70) | | 0.6 | |
| Platelet [10^3^/μl] | | 186 (156, 237) | | 272 (212, 336) | | 0.053 | |
| Albumin [g/dl] | | 3.85 (3.68, 4.20) | | 3.90 (3.50, 4.10) | | >0.9 | |
| NLR | | 2.1 (1.7, 2.5) | | 3.6 (2.9, 7.0) | | **<0.001** | |
| PLR | | 130 (93, 167) | | 260 (229, 317) | | **<0.001** | |
| SII | | 405 (335, 493) | | 977 (753, 2,072) | | **<0.001** | |

^1^ Shown as number with percentage or as median with interquartile range. *CNS* central nervous system; *G-CSF* Granulocyte-colony stimulating factor; *MRI* magnetic resonance imaging; *PET* Positron emission tomography; *CSF* cerebrospinal fluid; *CSI* cerebrospinal irradiation; *SI* spinal irradiation; *WB* whole brain irradiation; *NLR* neutrophil-lymphocyte count; *PLR* platelet-lymphocyte count; *SII* systemic immune-inflammation index.
